# CRISPR/Cas9基因编辑系统在肿瘤研究中的应用进展

**DOI:** 10.3779/j.issn.1009-3419.2015.09.08

**Published:** 2015-09-20

**Authors:** 超 刘, 志伟 李, 艳桥 张

**Affiliations:** 150081 哈尔滨，哈尔滨医科大学附属肿瘤医院内八科 Harbin Medical University Cancer Hospital, Harbin 150081, China

**Keywords:** CRISPR/Cas9, 基因编辑, 肿瘤基因, CRISPR/Cas9, Gene editing, Cancer genes

## Abstract

CRISPR/Cas9（Clustered Regularly Interspaced Short Palindromic Repeat/CRISPR-associated nuclease 9）基因编辑系统是基于古细菌抵御外源核酸入侵的免疫机制为基础开发出来的一种新型的基因编辑技术。相对于传统的基因编辑系统，该系统具有更加高效、操作简单、细胞毒性小等特点。目前，CRISPR/Cas9基因编辑技术已经在肿瘤研究的诸多方面中得到应用，包括肿瘤相关基因的功能研究、构建动物肿瘤模型、筛选肿瘤细胞表型及耐药相关基因以及肿瘤的基因治疗等诸多方面。本文就其在肿瘤研究中的应用进展进行综述。

## Abstract (en)

TCRISPR/Cas9 (Clustered Regularly Interspaced Short Palindromic Repeat/CRISPR-associated nuclease 9) gene editing system is a new type of gene editing technology developed based on the immune mechanism of archaea resisting the invasion of exogenous nucleic acid. Compared with traditional gene editing system, CRISPR/Cas9 system is more efficient, easier operating, and less cytotoxic. Currently, CRISPR/Cas9 gene editing technology has been applied to many aspects of cancer research, including research on cancer genes, constructing animal tumor models, screening tumor resistance-associated and phenotypic-related genes and cancer gene therapy. In this review, the application of the CRISPR/Cas9 system in tumor research were introduced.

肿瘤的发生和发展是一个多基因共同参与、涉及多条信号通路、缓慢而持续的过程^[1]^。在后基因组时代，得益于高通量测序技术和生物信息学技术的高速发展，研究者们从不同的肿瘤细胞中获得了大量的基因组信息。这些基因在肿瘤发生发展过程中，不同的基因在同一发展阶段，甚至同一个基因在不同的阶段都发挥着不同的生物学作用。如何探索这些基因与肿瘤的关系成为了研究者们面临的最重要的挑战。所以，对于肿瘤相关基因的功能研究，需要在不同的细胞分化阶段，对不同的基因表达进行有效的干扰，并以此为基础研究该基因对于肿瘤发生发展的影响。为此，研究者们迫切需要一个快速且有效的基因编辑技术，来人为地控制细胞内基因的表达水平。CRISPR/Cas9（Clustered Regularly Interspaced Short Palindromic Repeat/CRISPR-associated nuclease 9）基因编辑技术，相对于传统的基因编辑技术，如锌指核酸内切酶（zinc finger endonuclease, ZFN）^[2]^和类转录激活因子效应物核酸酶（transcription activator-like effector nuclease, TALEN）^[3]^以及小片段干扰RNA技术后，更进一步地填补了研究者们在此方面的空缺，并且从多个方向快速地推进了肿瘤研究的进展。

## 1 关于CRISPR/Cas9基因编辑技术的介绍

CRISPR/Cas系统是细菌和古细菌在不断进化的过程中产生的适应性的免疫防御机制，用来保护自身的基因组免受外源核酸如噬菌体、病毒等的干扰和破坏^[4]^。CRISPR/Cas系统作为一套适应性免疫系统，他应用CRISPR RNA（crRNA）以碱基互补的形式引导Cas蛋白识别入侵的外源基因组，并对其DNA进行剪切。根据Cas蛋白的序列和结构将CRISPR/Cas系统分为Ⅰ型、Ⅱ型和Ⅲ型^[5]^。其中Ⅰ型和Ⅲ型系统都需要多个Cas蛋白原件，而Ⅱ型仅仅需要Cas9蛋白^[6]^。Ⅱ型CRISPR/Cas系统（即CRISPR/Cas9系统）已经在化脓链球菌^[7]^和奈瑟球菌^[8]^内重点研究，并且已被发展成为一套理想的程序化的基因编辑工具。

### 1.1 CRISPR/Cas9系统的组成结构

#### 1.1.1 Cas9核酸内切酶（CRISPR-associated nuclease 9）

Cas9核酸内切酶是Ⅱ型CRISPR/Cas系统最必不可少的组成元件，他能够以针对具有特异性DNA序列的DNA进行双链剪切^[5]^。Cas9核酸内切酶是一个大的多结构域蛋白，其中两个核酸内切酶结构域分别为：位于N端的RuvC核酸内切酶样的结构域，以及一个在中部有类似HNH核酸酶的活性的结构域。在体外实验中已经证实，化脓链球菌内的Cas9蛋白可以对靶DNA造成双链断裂切口。其中，HNH核酸内切酶结构域完成对与crRNA互补链的剪切，而RuvC样核酸内切酶切割另外一条非互补链^[9]^。有趣的是，当对这两个内酸酶位点进行突变后，并不影响CRISPR/Cas9复合体与外源性间隔序列位点的结合。另外，在间隔序列旁有一段保守的短的核酸序列，如NGG、NGGNG、NAAR和NNAGAAW，即间隔序列临近基序—PAM结构（protospacer adjacent motif），对于Cas9蛋白进行结合和剪切都是至关重要的^[10]^。不同的微生物需要不同的PAM结构，但是对于Cas9蛋白需要识别特异的PAM结构的机制仍然是未知的^[11]^。

#### 1.1.2 反式激活crRNA（transactivating crRNA, tracrRNA）

tracrRNA是CRISPR/Cas9系统第二个组成的部分，他是一个非蛋白编码RNA，主要用来完成crRNA的成熟和之后的DNA剪切^[12]^。在化脓性链球菌中，tracrRNA由两个起始位点转录产生分别长度为171个核苷酸和89个核苷酸序列前体tracrRNA，随后两者再进一步加工成为75个核苷酸的成熟体tracrRNA。tracrRNA前体的一部分序列能够与crRNA前体互补，有助于crRNA的成熟^[7]^。

#### 1.1.3 CRISPR RNA（crRNA）

CRISPR RNA携带着CRISPR/Cas系统中间隔序列的序列，负责捕捉外源性的DNA序列，起到复合体定位的功能。在化脓链球菌中，前体crRNA（pre-crRNA）是一条长度为511个核苷酸的RNA，他包括一个前导区和一系列重复-间隔-重复的序列组成^[7]^。前体crRNA需要借助tracrRNA、RNaseⅢ以及Cas9蛋白经过两步剪切才能成为成熟的crRNA，其长度约为39个-42个核苷酸序列，其中包括一个独一无二的20个核苷酸的间隔序列和一个19个-22个核苷酸长度的重复序列。这些序列可以特异性地识别到入侵基因组的剪切位点，指导CRISPR/Cas9系统进行基因编辑。

### 1.2 CRISPR/Cas9系统的作用过程

#### 1.2.1 CRISPR序列的捕获与记忆

在初始阶段，噬菌体或外源性的核酸首次入侵细菌时，CRISPR系统需要把外源核酸上的一部分序列即原间隔序列整合到宿主基因组上成为间隔序列，然后这部分储存在间隔序列内的信息再用于抵抗外源基因的干扰^[13]^。所以起始阶段属于CRISPR系统行使功能的适应阶段。大量研究发现所有的CRISPR系统中都含有Cas2基因编码区，表明Cas2在CRISPR系统作用的过程中起着非常重要的作用，研究结果也表明Cas2参与了新的间隔序列的形成^[5]^。同时，研究发现在CRISPR基因座上临近间隔序列的附近有几个非常保守的核苷酸的序列，称为间隔序列临近基序—PAM。不同的细菌和古细菌需要不同的PAM结构，在化脓链球菌中的Ⅱ型CRISPR系统的PAM结构序列为NGG。PAM结构的作用是将间隔序列定位于入侵的噬菌体或质粒的DNA序列中，即宿主对入侵的外源核酸进行扫描，在其DNA序列中定位若干个PAM，并将PAM结构旁的序列定义为新的原间隔序列并被剪切后陆续的整合到CRISPR系统新合成的两个重复序列之间。以此完成对外源核酸序列的捕获和记忆。

#### 1.2.2 CRISPR系统的转录与转录后加工

CRISPR系统的表达包括tracrRNA和crRNA的转录与成熟过程。重复序列和间隔序列在前导序列的启动下进行转录形成pre-crRNA，pre-crRNA通过剪切形成成熟的crRNA。以化脓链球菌内的Ⅱ型CRISPR系统为例^[7]^，tracrRNA由两个起始位点转录产生长度分别为171个核苷酸和89个核苷酸序列的前体tracrRNA，随后二者再进一步加工成为75个核苷酸的成熟体tracrRNA。前体tracrRNA的一部分序列能够与crRNA前体互补，有助于crRNA的成熟。Pre-crRNA需要借助tracrRNA、RNaseⅢ以及Cas9蛋白经过两步剪切才能成为成熟的crRNA，crRNA长度约为39个-42个核苷酸序列，其中包括一个单独的20个核苷酸的间隔序列和一个19个-22个核苷酸长度的重复序列。正常情况下细菌中的CRISPR的表达水平比较低。但是，当噬菌体或外源质粒再次入侵时，CRISPR会被迅速地诱导表达上调。

#### 1.2.3 CRISPR/Cas9系统对外源核酸进行剪切

成熟后的crRNA与tracrRNA会有部分互补的区域，从而形成双链的RNA，再与Cas9蛋白共同形成核糖核蛋白复合物。crRNA上的间隔序列会特异性地结合到外源核酸与其互补的序列上，然后解开DNA双链，形成R环使crRNA与互补链杂交，另一条链保持游离的单链状态，然后由复合体中Cas9蛋白中HNH结构域剪切与crRNA的互补DNA链，RuvC结构域剪切非互补链。在CRISPR/Cas9系统复合体的作用下，外源性的核酸会在特定的位置被剪切，形成双链断裂切口（double strand break, DSB），从而完成细菌对外源核酸的干扰^[9]^。

### 1.3 利用CRISPR/Cas9系统进行基因编辑的作用过程

基于对Ⅱ型CRISPR/Cas系统即CRISPR/Cas9系统的研究，研究者们已经将该套系统改造成为一套理想的程序化的基因编辑工具。Cas9介导的基因编辑依赖两个连续的步骤：首先，Cas9核酸内切酶在crRNA的介导下对基因组DNA进行剪切；然后，DNA的DSB会被细胞内天然的DNA修复系统进行修复^[14]^。

首先利用CRISPR/Cas9系统剪切特定的DNA序列，需要三个重要的元件，包括：Cas9蛋白、tracrRNA、依据需要设计的特异性地crRNA。将这三个元件转染到目标细胞内，当该系统表达时，其tracrRNA会与crRNA形成双链RNA并定向地与目标DNA结合，同时Cas9蛋白会对目标DNA的正链和反链进行剪切，形成DSB，随后细胞会启动天然的DNA修复系统，完成DNA的修复。研究发现，将pre-crRNA和tracrRNA构建成一个融合RNA（single-guide RNA, sgRNA）来模拟成熟的crRNA和tracrRNA，同样可以与Cas9共同作用特异地切割目的DNA，从而将CRISPR/Cas9系统简化成Cas9蛋白和sgRNA两个组分。在大多情况下，Cas9蛋白和sgRNA两个组分切割DNA的效率比Cas9蛋白、pre-crRNA和tracrRNA三个组分的效率高。

DSB修复的方式有两种，一种是非同源末端连接（native nonhomologous end joining, NHEJ）^[15]^，这种修复方式往往会导致随机的小片段的缺失或者插入（indels）以此来导致靶基因的故障，但这种编辑方式是不能够进行精确控制的；另一种是同源重组介导的修复（homology-directed repair, HDR）^[16]^，在双链DNA断点产生后，同时引入模板DNA序列，这部分序列根据需要，插入特定的一段序列（knock in）、删除特定的序列（knock out）或突变特定的碱基或序列，这时候通过同源重组的作用进行断链修复，就可以把需要编辑的序列引入到特定的位点，以此完成精确的基因编辑。

## 2 CRISPR/Cas9基因编辑技术在肿瘤研究中的应用

CRISPR/Cas9系统作为一个新兴的基因编辑技术，目前在生物学领域内，对该系统的应用模式主要集中在以下三个方向：①利用CRISPR/Cas9基因编辑技术定点编辑目标基因，目前已广泛应用于各种真核、原核生物的基因工程方面^[17]^；②基于CRISPR/Cas9系统的基因组规模的基因编辑，再结合高通量测序技术筛选与表型相关基因的技术^[18]^；③利用灭活核酸酶活性后的Cas9（dCas9），将其改造成为一个利用RNA引导的归航装置，并通过改变与dCas9相融合的效应器来扩展该系统更加广泛的用途^[19]^。目前，在肿瘤的研究中CRISPR/Cas9基因编辑技术也被广泛应用，并获得了许多令人欣慰的成果。

### 2.1 研究基因在肿瘤发生发展相关机制中的应用

#### 2.1.1 白血病相关基因功能研究

先天性和获得性的7号染色体长臂缺失，常见于骨髓异常增生综合症和急性粒细胞白血病中，并且往往提示与不良预后相关^[20]^。但是对于位于7号染色体长臂上的相关抑癌基因的功能的研究目前尚不清晰。组蛋白H3K4三甲基化转移酶（MLL3）是7号染色体上的一个抑癌基因，他属于MLL蛋白家族，可以对H3K4位置的赖氨酸进行甲基化，进而影响相关基因的转录活性^[21]^。目前，已经发现该基因在结肠癌、髓母细胞瘤、乳腺肿瘤、白血病等疾病中广泛存在^[22, 23]^。利用CRISPR/Cas9基因编辑技术对可以完成对单条或者两条等位基因的编辑。有研究^[24]^利用该技术对*MLL3*基因进行编辑，并获得了该基因表达水平下调至野生型的50%水平的小鼠模型。以此为基础，配合其他因7号染色体长臂缺失的事件，最终促进白血病的发生。有趣的是，MLL3半数抑制后导致的白血病，与人类7号染色体缺失或者7号染色体长臂缺失后导致的白血病一样，都是对常规化疗的敏感性欠佳，而对BET抑制剂JQ1有较好的疗效。因此，研究者们认为他们构建的小鼠模型成功地证实了*MLL3*基因是7号染色体长臂上的一个单倍剂量不足的抑癌基因，并且为这种严重的疾病的治疗提供了一个新的策略。

#### 2.1.2 实体瘤相关基因功能研究

CRISPR/Cas9技术同样运用到了对实体瘤的研究中，以一项关于直肠癌与*PIK3R1*基因关系的研究为例^[25]^。在这项研究中，研究人员利用CRISPR/Cas9技术在直肠癌细胞系水平上敲除*PIK3R1*基因，之后分别检测该敲除细胞系与野型细胞系在上皮细胞间质化（epithelial-to-mesenchymal transition, EMT）及细胞增殖、肿瘤细胞干细胞特性等方面的变化，以此证明PIK3R1基因具有调节直肠癌细胞的侵袭转移及肿瘤干细胞特性的功能。

### 2.2 利用CRISPR/Cas9基因编辑技术建立小鼠肿瘤模型

肿瘤模型的建立，为研究肿瘤发生、发展的机制和对治疗探索提供了极大的帮助。小鼠模型是目前可以整合基础和临床肿瘤研究的理想载体，已广泛应用于肿瘤研究的各个领域。小鼠肿瘤模型按照建立的方法可分为以下几类^[26]^：①自发和诱发小鼠模型；②移植型小鼠模型；③基因敲除小鼠模型；④转基因小鼠模型等。

基因敲除小鼠肿瘤模型是指利用基因敲除的方法去除抑制肿瘤发生相关的基因，从而诱导小鼠肿瘤发生的小鼠模型^[27]^。这对于认识单一基因或数个基因在肿瘤发生中的作用而言，是一个理想模型。在过去30年中，基因编辑技术的开发和进步，同样促进了用于研究人类恶性肿瘤特点的小鼠肿瘤模型。2014年，在*Nature*和*Cell*杂志上分别介绍了利用CRISPR/Cas9基因编辑技术构建的小鼠肿瘤模型。

#### 2.2.1 小鼠肝癌模型

利用小鼠模型对癌症基因的研究，传统上多是采取在受精卵水平上进行基因编辑，然后将该编辑后的受精卵培养成为成鼠^[28]^。在这篇*Nature*的文章中，张锋的研究团队介绍了一种新的构建小鼠基因敲除肿瘤模型的方法，即利用CRISPR/Cas9系统，在野型小鼠的体内进行基因编辑^[29]^。研究者们利用小鼠尾静脉液压注射技术，将表达有Cas9核酸内切酶和一个sgRNA的DNA质粒注射入小鼠肝脏，同时或者单独对小鼠的*Pten*和*P53*抑癌基因进行编辑。利用CRISPR/Cas9系统介导*Pten*突变后的小鼠，会导致Akt磷酸化水平的升高，以及过多的脂质在小鼠肝脏细胞内积聚。与此同时，研究者们同样利用传统的基因编辑技术——Cre-loxP重组酶系统模拟了小鼠敲除Pten和p53后导致的肝癌模型的表型，与前者表型一致。通过对小鼠肝脏和肿瘤组织的DNA测序，发现在肿瘤细胞中存在抑癌基因Pten和p53双等位基因插入或者缺失的突变。此外，研究者们还利用CRISPR系统，将Cas9表达质粒、靶向β-catenin的sgRNA以及能够诱导β-catenin突变的寡链核苷酸共同注射入小鼠体内，导致小鼠肝细胞内发生突变后的β-catenin蛋白发生核移位，最终导致小鼠肝脏的癌变。这项研究揭示了新兴的基因编辑技术CRISPR/Cas9系统，在小鼠肝细胞内直接进行抑癌或者原癌基因编辑的可行性，并且提供了肝癌模型构建的新方法，同时为基因组学的研究提供了新的思路。

通过该项试验，研究者们证实了利用CRISPR/Cas9系统在成年小鼠的肝脏内进行抑癌基因和原癌基因的编辑。这种方法避开了在胚胎干细胞水平上进行的编辑，以及饲养杂合体小鼠的繁琐。另外，借助腺病毒的方式、更加高效的sgRNA以及更长的相互匹配的模板，这些比之前更加有效的递送技术，也许能够提高该项技术的编辑效率，并有机会扩展更多的器官适用于此。总之，这项研究扩展了CRISPR/Cas9技术的应用，使之更加广泛，并且推动了小鼠肿瘤模型研究的发展。

#### 2.2.2 小鼠肺癌模型

在利用CRISPR/Cas9系统构建小鼠肝癌模型后不久，张锋及其团队的研究人员又成功利用该技术构建出了小鼠肺癌模型^[30]^。与之前构建小鼠肝癌模型的方法不同的是，研究者们首先构建出了*Cas9*基因敲入小鼠。这一模型小鼠简化了CRISPR/Cas9系统在体内进行基因编辑的步骤，并提高了编辑效率。利用这一新型的Cas9小鼠，研究人员成功编辑了小鼠体内的各种细胞类型的多个基因，并成功构建出了小鼠肺腺癌模型。

为了扩展CRISPR/Cas9技术在体内进行基因编辑的应用，研究者们利用Cre-loxP系统将Cas9基因敲入小鼠体内，构建出一个细胞内能够稳定表达Cas9核酸内切酶的小鼠（Cas9小鼠），这样便克服了向成鼠体内导入Cas9蛋白的困难。随后，研究者们分别在体内和体外，利用腺病毒、慢病毒以及颗粒介导的形式对小鼠的神经细胞、免疫细胞和内皮细胞内转导入sgRNA。利用这些小鼠，研究者们选择了三个基因，即*KRAS*、*p53*和*LKB1*，当这些基因发生突变时能够诱导肺腺癌的发生。然后，利用腺病毒的形式向该Cas9小鼠中，导入*p53*和*LKB1*基因的sgRNA以及利用同源重组的方式来编辑*KRAS*基因，致其发生突变，最终导致在小鼠体内出现肺腺癌的病理变化。结合研究人员的研究结果，证实了Cas9小鼠模型在生物学和疾病建模中的广泛应用。

目前，该小鼠模型已经被提供给全世界范围内的多家科学团队，研究人员们正在利用该小鼠模型继续进行基因组学的研究。

#### 2.2.3 小鼠白血病模型

近年来基因组测序研究的结果显示，在人类恶性肿瘤的发生过程中，往往存在4个甚至更多个基因的突变，但是利用传统的小鼠模型育种方法，很难在小鼠模型中模拟出这种基因突变的复杂性。2014年6月在*Nature*子刊上刊登了一篇文章介绍了利用CRISPR/Cas9基因编辑技术克服了这一限制^[31]^。来自美国哈佛医学院的研究人员仍然利用慢病毒转导的方式，将sgRNA和Cas9共同递送入同一只小鼠的造血干细胞中，对其中的5个基因同时进行编辑，从而导致小鼠骨髓恶性肿瘤的发生。因此而获得的该急性粒细胞白血病模型小鼠能够成功地与白血病患者中出现的转录因子、信号通路的相关的细胞因子等基因突变相匹配。这项研究结果向我们展示了，利用慢病毒转导sgRNA和Cas9的系统进行基因编辑，能够被用来进行体内的多基因突变肿瘤模型的构建，从而更好地模拟人类疾病的复杂性。

### 2.3 利用CRISPR衍生技术筛选与肿瘤表型相关的基因

得益于CRISPR/Cas9技术在基因编辑方面的高效性，在同一个细胞系中在基因组规模上，同时进行多个基因的编辑成为了可能。因此，研究人员们可以在基因组规模上，筛选出细胞内的哪些基因和研究者们感兴趣的表型相关。利用慢病毒转导的方式可以同时向细胞内转导入成千上万个靶向不同基因的sgRNA。最近，多篇文章的发表，证实了这种筛选方式在人类细胞中阴性和阳性的筛选能力。以往在基因组规模上进行这种功能缺失的筛选都是应用的RNAi的方法^[32]^，但是这种方法仅仅能够让基因的表达水平下调，并且有着较为严重的脱靶效应。相反，Cas9介导的sgRNA文库筛选系统已经被证实具有更高的筛选敏感性，而且几乎可以靶向调节任何一段DNA序列^[33-35]^。在将来利用sgRNA筛选文库可能会扩展到对非编码基因序列的筛选中^[34]^。

另外，随着dCas9（endonuclease-deficient Cas9即核酸内切酶活性缺失的Cas9）技术的发展及应用，通过将不同的功能原件与其融合，基因筛选的方式可能不再只是功能缺失表型的筛选^[18]^。例如，将dCas9与转录激活原件融合，就可以进行功能获得表型的基因筛选。

#### 2.3.1 利用GecKO文库筛选与黑色素瘤耐药相关的基因

2014年*Science*和*Nature*杂志上连续报道了3篇关于利用Cas9技术在人类细胞中进行基因筛选的文章^[18, 36-38]^。其中，张锋及其团队^[18]^构建出了靶向人类基因组中18, 080个基因的庞大的sgRNA文库，并命名为GecKo（genome-scale CRISPR-Cas9 knockout）Library。在这个文库中，设计人员们针对每一个基因设计了3个-4个sgRNA，即整个文库包含了64, 751个不同的sgRNA。结合慢病毒转导和高通量测序的方法，最终完成该文库对表型相关基因的筛选。

首先，研究人员利用该文库对黑色素瘤细胞和多能干细胞的必需基因进行了阳性和阴性筛选。接下来，研究者们在黑色素瘤A375细胞系中利用GecKo文库筛选了黑色素瘤细胞中与vemurafenib（一种RAF抑制剂）耐药相关的基因。通过高通量测序后的对比，研究人员最终得到了6个高度可疑的候选基因，其中*NF1*和*MED12*基因已经在以前的研究中被证实与该药物耐药相关，另外还有基因*NF2*、*Cul3*、*TADA2B*和*TADA1*，这4个基因是新发现的可能与该药物耐药相关的基因。

#### 2.3.2 利用GecKO文库筛选与肺癌转移相关的基因

2015年2月，张锋教授及其研究团队^[39]^再接再厉，在*Cell*杂志上发表了最新的研究成果：利用CRISPR/Cas9技术改造的GecKo文库在小鼠体内筛选出和肿瘤细胞生长和转移相关的基因。在这项研究中，研究人员选择了一个非小细胞肺癌的细胞系——KPD，然后借助慢病毒载体，将融合有GFP的Cas9基因序列整合到该细胞系基因组中，构建出Cas9-GFP-KPD细胞系。接下来研究人员将之前构建的mGecKoa文库（包括67, 405个sgRNA分别靶向20, 611个基因和1175个microRNA）仍然以慢病毒的形式转导入Cas9-GFP KPD细胞系中，并在体外培养一周。之后，将该转导有Cas9及sgRNA文库的细胞系及未经文库干扰的细胞系分别植入裸鼠体内。将小鼠培养一段时间后，比较两组小鼠肿瘤生长及转移情况，结合高通量测序技术，得出差异性基因，提示这些基因与非小细胞肺癌生长和转移相关。

#### 2.3.3 CRISPR介导的基因表达调节筛选白血病的抑癌基因

对于哺乳动物来说，细胞内同一个基因的表达水平在不同的细胞类型以及疾病发展的不同阶段是不一样的，但是研究者们对这种转录水平的变化的了解却是相对落后的。有文献^[33]^展示了一项新的系统，能够用来系统地研究细胞内基因在转录水平下调和上调时的变化。首先，研究人员通过将融合有转录抑制结构域的dCas9（即CRISPRi技术），靶向到基因序列启动子附近的不同位置，从而达到不同的干扰效果。并以此建立标准，最终能够可控的、在最小脱靶的情况下将基因表达水平下调90%-99%。然后，将转录激活结构域融合到dCas9上，来激活基因的表达（CRISPRa技术）。结合这两项技术，最终可以把基因的表达水平调节在0.1倍-10倍的范围内。利用这一标准，研究人员构建出了一个基因组规模的CRISPRi和CRISPRa文库，并利用此文库进行基因的筛选。通过白血病细胞生长为基础的筛选，可以筛选出细胞生长的必需基因、肿瘤的抑癌基因和调节细胞分化的基因。另外，研究者们还利用此文库进行了细胞对一种霍乱和白喉融合毒素的耐药和敏感表型的基因筛选，并以此建立了一个假想的信号通路图。基于这些研究结果，设计者们认为，CRISPRi和CRISPRa文库可以作为一个有力的工具，提供丰富且完整的信息以探索复杂的信号通路。

### 2.4 在肿瘤基因治疗中的探索应用

基因治疗是指通过特殊的方法改变患者的遗传物质，将人的正常基因或者有治疗作用的基因通过一定方式导入患者的靶细胞，直接针对患者的异常基因本身进行的治疗，从而达到治疗疾病的目的^[40]^。肿瘤的基因治疗具有特异性、安全性、有效性的特点，是目前肿瘤治疗研究领域的热点。

#### 2.4.1 膀胱癌基因治疗研究中的新思路

在全世界范围内，膀胱癌都是一个非常常见的泌尿系统肿瘤。传统上针对于膀胱癌的治疗多是化疗或者放疗，但是这些治疗往往会杀死除肿瘤细胞以外的正常细胞，从而导致严重的副作用和治疗失败^[41]^。而针对于人类肿瘤相关基因的基因治疗策略，是一个非常具有前景的膀胱癌治疗方式。利用这种治疗策略，需要启动凋亡和细胞毒性的基因，而且只有肿瘤细胞才会表达活跃的启动子进行基因编辑，从而特异性地诱导肿瘤细胞的凋亡或坏死。但是在肿瘤细胞中寻找这种特殊的启动子是很困难的，因为某些基因虽然在某个器官的肿瘤细胞内表达活跃，这个基因也有可能在其他不同组织的正常细胞内也会表达水平相对活跃。这就造成了以下困难：首先是这种治疗策略的低效性；其次就是很多特殊的肿瘤基因启动子往往是低表达的。因此，现在亟需一个能够提高肿瘤基因治疗效率和可行性的方法。

在这项实验中，研究人员就巧妙地运用了CRISPR/Cas9基因编辑技术和逻辑通路的原理，解决了上述问题^[42]^。在这里，研究人员设计出一种基于CRISPR/Cas9系统的逻辑通路，输入端是由两个启动子组成，在细胞系内只有当这两个输入端口同时被激活时，输出端的基因才会被激活。首先研究者分别将肿瘤特异性高表达启动子hTERT与sgRNA融合，将膀胱的组织特异性高表达启动子hUPII与表达Cas9的基因融合。当细胞系同时存在这两个启动子高表达时，才能激活CRISPR/Cas9系统，对其下游基因进行编辑，从而激活输出端基因。该实验中，分别利用荧光素酶报告基因，与细胞凋亡相关的BAX基因，与细胞生长相关的p21基因，与肿瘤细胞转移相关的*E-cadherin*基因作为输出基因。实验证明，只有在膀胱癌的细胞系中，逻辑通路才会激活这些输出基因，从而诱导肿瘤细胞表型的进一步变化。这项研究为肿瘤基因治疗的研究提供了新的思路。

#### 2.4.2 治疗HPV感染导致的宫颈癌的研究

高危型人乳头状瘤病毒癌基因（E6和E7）表达异常是宫颈癌发病的重要原因^[43]^。所以，这两个病毒癌基因也被视为重要的治疗靶点。为了开发特异性的针对HPV感染相关肿瘤的基因治疗的策略，研究人员基于CRISPR/Cas9技术建立了靶向编辑E6、E7基因的编辑系统，并将该系统转染入感染HPV-16病毒阳性的宫颈癌细胞系——SiHa细胞^[44]^。结果表明，利用CRISPR/Cas9系统进行E6、E7基因及其启动子的编辑后，会导致p53和p21蛋白在细胞内的积累，从而显著降低宫颈癌细胞系在体外的增殖能力。随后，研究人员进行了裸鼠的皮下接种肿瘤细胞的实验，构建皮下植瘤的动物模型。研究发现，经CRISPR/Cas9系统靶向编辑E6、E7基因后的肿瘤细胞接种的小鼠体内，肿瘤的生长受到显著抑制。这项研究结果为CRISPR/Cas9系统在靶向编辑高危型HPV致癌基因的基因治疗中的应用提供了证据；并且作为一种新的治疗策略，在宫颈癌和其他HPV相关癌症的治疗中提供了新的思路。

### 2.5 在肿瘤的其他研究方面中的应用

#### 2.5.1 利用CRISPR/Cas9系统构建肿瘤相关染色体易位模型

人类肿瘤相关的染色体易位往往是通过两个DSB产生的非同源染色体的异常接合形成^[45]^。在研究白血病和肉瘤发病的初始机制时，需要一个有效的方法能够精确的重现这种肿瘤相关的染色体易位。有研究人员^[46]^利用gRNA介导的CRISPR/Cas9系统，在体外特定的位点构建出这种癌症相关的染色体易位。利用这一方法，Rodriguez-Perales成功地在人类细胞系和原代细胞中模拟出与急性骨髓性白血病和尤因氏肉瘤高度一致的模型。利用荧光原位杂交技术（fluorescence *in situ* hybridization, FISH）和分子分析对这些编辑后的细胞的融合基因的mRNA和蛋白水平进行检测，显示出CRISPR-Cas9方法的可靠性和精确度，这为染色体易位相关肿瘤的研究提供了一个有力的工具。

#### 2.5.2 在肿瘤相关miRNA研究中的应用

microRNA能够调节血管生成的平衡^[47, 48]^。最近的一项研究^[49]^表明，当所有microRNA枯竭，会导致肿瘤的血管生成受到抑制。研究人员通过敲除Dicer1形成microRNA缺陷的肿瘤。这些肿瘤处于高度缺氧状态，但是其血管生成不良，提示了其血管生成相关信号的不足。通过基因表达谱检测分析，由于microRNA枯竭，导致了血管生成基因的表达显著下调。但是，在这一背景下，FIH1（HIF-1抑制因子）却是激活的状态，进而能够抑制HIF的转录活性。随后，在microRNA缺陷的细胞系中利用CRISPR/Cas9系统敲除*FIH1*基因，这时，HIF-1的转录活性、血管内皮生长因子的产生、肿瘤缺氧和肿瘤血管生成的情况都会较之前有相反的变化。继续利用CRISPR/Cas9系统对*FIH1*基因非编码区中的microRNA的结合位点进行编辑，则会出现FIH1蛋白的表达正常，但是细胞对缺氧环境的反应能力却未能恢复。这些研究结果表明，microRNA能够通过抑制*FIH1*基因来促进肿瘤对低氧和血管生成的反应。

#### 2.5.3 构建肿瘤类器官

肿瘤类器官能够复制原发肿瘤的一些关键特性，这种“类器官”培养物适用于大规模的药物筛查来检测与药物敏感性相关的一些遗传改变，为采用个体化治疗改善癌症患者的临床结局铺平了道路。目前，人们主要还是利用培养皿中的二维细胞系或是在小鼠模型中开展抗癌药物筛查。肿瘤类器官相对于细胞系更加近似人类肿瘤，且比小鼠模型更节省时间和资源，为研究人员提供了现有方法之间的一个折中策略。利用CRISPR/Cas9基因编辑技术，Keio大学的研究团队建立了源自正常肠道上皮的大肠癌类器官^[50]^。这项发表在*Nature Medicine*杂志上的研究，实验人员通过CRISPR/Cas9系统向人类正常肠道上皮组织内引入了肿瘤抑制基因*APC*、*SMAD4*和*TP53*突变，以及癌基因*KRAS*和*PIK3CA*突变。他们发现，表达全部五种突变的类器官，不依赖干细胞巢的因子就能在体外生长，而且移植到小鼠肾包膜下会形成肿瘤。进一步研究表明，这种类器官注射到小鼠脾脏之后会发生微转移，但癌细胞并不能在肝脏站稳脚跟。而源自人类腺瘤的类器官（染色体不稳定）在移植后会形成大的转移集落。这项研究指出，激活通路突变可以在肿瘤微环境中维持肿瘤干细胞特性，但结直肠癌细胞的侵袭性行为还需要细胞内出现更多的分子损伤。

## 3 小结

从来没有哪项生物学技术能像CRISPR/Cas9系统这样风靡整个科学界，赢得了全世界科学家的热切关注，并且迅速成为基因编辑的主要技术手段。同样，随着该项技术的广泛应用，CRISPR/Cas9基因编辑技术存在的问题也暴露出来，其中表现最为突出的就是这种基因编辑的脱靶效应^[51]^。

脱靶效应是指基因编辑系统对目标靶点以外的基因片段进行修饰。由于CRISPR/Cas9系统的向导RNA片段与目标DNA片段只需要20个核苷酸进行匹配，所以这段RNA也极有可能会与目标外的其他片段发生结合。美国麻省总医院的Joung博士的团队研究^[52]^发现这种脱靶位点的突变的确存在，其突变率甚至较目标靶点的突变效率相同甚至更高。因为这种脱靶效应干扰了细胞内其他基因的稳定性，由此带来的非预期的细胞生物学行为和问题也随之而来，包括基因敲除效率降低、严重的细胞毒性出现，以及给肿瘤基因治疗带来的挑战。面对这些问题，研究者们探索了一系列的方法来改善这种情况，如设计特异程度较高的sgRNA；除靶标外，尽量避免在基因组中sgRNA后紧随PAM结构；突变Cas9核酸酶的RuvC-like结构域或HNH结构域，将CRISPR/Cas9系统改造为切口酶，激活细胞的同源重组修复机制，对降低细胞毒性具有一定作用。

目前，随着基因组信息不断积累，人类对于基因功能的研究远远滞后于发现新的基因的速度。在这个背景下，CRISPR/Cas9基因编辑系统作为新兴的基因编辑技术一经发表，即获得广泛的关注。这一高效而简单的基因编辑技术，为更多的实验平台提供了有力的研究基因功能的武器。肿瘤研究作为多年来生物学的研究热点，也从中获益匪浅。从单一肿瘤相关基因功能的研究到小鼠肿瘤模型的构建，再到Cas9衍生技术用于肿瘤相关表型基因的筛选以及在肿瘤基因治疗中的探索。在短短的2年时间内，CRISPR/Cas9基因编辑技术势如破竹地解决着肿瘤研究中的一个又一个难题。作为21世纪最为杰出的生物学发现之一，这项技术势必会在生物学基因研究领域内掀起一场新的革命，也注定会推进肿瘤研究的快速发展，并且为更多的肿瘤患者带去新的希望。
